# Pacemaker Lead-Associated Innominate Vein Thrombosis Despite Non-vitamin K Oral Anticoagulant (NOAC) Therapy: An Incidental CT Finding

**DOI:** 10.7759/cureus.104717

**Published:** 2026-03-05

**Authors:** Neeraj Joshi, Rachel Hallam, Zaw Nyein Aye, Harriet Guthrie, Md Mahmud Hasan

**Affiliations:** 1 Cardiology, East Kent Hospitals University NHS Foundation Trust, Queen Elizabeth The Queen Mother (QEQM) Hospital, Margate, GBR; 2 Medicine, East Kent Hospitals University NHS Foundation Trust, Queen Elizabeth The Queen Mother (QEQM) Hospital, Margate, GBR

**Keywords:** anticoagulation, brachiocephalic (innominate) vein thrombosis, case report of educational intervention, device-related thrombosis (drt), incidental radiological finding, noacs, permanent pacemaker (ppm) complication, warfarin

## Abstract

Venous thrombosis occurring despite therapeutic non-vitamin K oral anticoagulant (NOAC) therapy represents a diagnostic and management challenge, particularly in patients with indwelling cardiac implantable electronic devices. Pacemaker-associated central venous thrombosis is a recognized but often underdiagnosed finding due to its frequently asymptomatic presentation. We report the case of an older adult with a permanent pacemaker implanted approximately 10 months earlier who was undergoing evaluation for unexplained weight loss, in whom cross-sectional imaging incidentally revealed left innominate vein thrombosis without clinical features of venous obstruction. The patient was receiving full-dose NOAC therapy for atrial fibrillation, with good adherence and no alternative provoking factors identified. This case highlights the importance of recognizing venous thrombosis in patients with cardiac implantable electronic devices despite therapeutic anticoagulation and supports a careful, mechanism-based approach to evaluation and management in similar situations.

## Introduction

Upper extremity and central venous thrombotic events are recognized findings in patients with cardiac implantable electronic devices (CIEDs). Transvenous pacing leads remain within the venous system and may be associated with local venous changes, including endothelial interaction and altered flow dynamics, which in some individuals may contribute to venous stenosis or thrombosis [1,2]. However, it is important to distinguish venographic abnormalities from clinically significant thrombosis, as many imaging-detected venous changes remain asymptomatic.

Earlier venographic studies in patients with pacemaker or implantable cardioverter-defibrillator leads have reported venous abnormalities in 23%-64% of patients, the vast majority of which are clinically silent [1]. In a prospective venography study performed during generator replacement, venous obstruction of varying severity was identified in approximately 25% of patients, including complete occlusion in 9%, severe stenosis in 6%, and moderate stenosis in 10% [2]. More recent data provide further insight into clinically relevant thrombotic events. A systematic review and meta-analysis reported a symptomatic upper extremity deep vein thrombosis (UEDVT) incidence of approximately 0.9 per 100 person-years, while the prevalence of asymptomatic venous occlusion was approximately 8.6% beyond the early post-implantation period [3].

Catheter-related thrombosis and pacemaker lead-associated thrombosis share overlapping mechanisms but differ in clinical context and implications for long-term venous access [4,5]. Although transvenous leads may contribute to local venous changes that predispose to thrombosis in some individuals, routine prophylactic anticoagulation solely to prevent device-related thrombosis is not recommended, as most venographic abnormalities remain clinically silent and current guidelines do not support anticoagulation in the absence of another indication [3,6].

Management strategies for lead-associated thrombosis are generally extrapolated from evidence for UEDVT. Contemporary venous thromboembolism guidance recommends anticoagulation for three to six months, with treatment duration individualized according to symptom burden, thrombus extent, and ongoing risk factors such as indwelling intravascular devices [6,7]. Direct oral anticoagulants (DOACs) are increasingly used for UEDVT and have demonstrated comparable efficacy and safety to vitamin K antagonists in observational studies and meta-analyses [8,9]. However, evidence specifically addressing pacemaker lead-associated thrombosis remains limited. When thrombosis is identified during therapeutic anticoagulation, some clinicians may consider transitioning to a vitamin K antagonist such as warfarin to allow international normalized ratio (INR)-guided anticoagulation monitoring, although this approach is largely based on extrapolated venous thromboembolism literature rather than device-specific randomized trials [6-9].

We report the case of an older adult with a permanent pacemaker who was already receiving therapeutic non-vitamin K oral anticoagulant (NOAC) therapy for atrial fibrillation, in whom cross-sectional imaging performed during evaluation for unexplained weight loss incidentally revealed left innominate vein thrombosis in the absence of clinical signs of venous obstruction.

## Case presentation

An elderly man in his early 80s presented with a two-year history of unintentional weight loss following a previous cholecystectomy and inguinal hernia repair. The weight loss was initially gradual and was not associated with gastrointestinal red-flag symptoms such as dysphagia, gastrointestinal bleeding, altered bowel habits, or persistent abdominal pain. Serial weight measurements demonstrated progressive weight loss from 80 kg in 2016 (BMI 27.7 kg/m²) to 66.6 kg in May 2025 (BMI 23 kg/m²) and 57.9 kg in February 2026 (BMI 20 kg/m²). As part of the initial evaluation for weight loss, the patient’s primary care physician arranged a computed tomography scan of the chest, abdomen, and pelvis (CTCAP, Video 1) approximately one year earlier, prior to pacemaker implantation. That study demonstrated no evidence of malignancy or metastatic disease.

**Video 1:** A contrast-enhanced CTCAP performed one year prior demonstrated normal opacification of the left innominate without evidence of venous obstruction. Contrast-enhanced CTCAP performed one year prior showing normal contrast specification of the left innominate vein without evidence of venous obstruction. CTCAP: computed tomography scan of the chest, abdomen, and pelvis

Because of ongoing weight loss, the patient’s primary physician arranged a repeat CTCAP (Video 2) through the urgent two-week suspected cancer pathway as an outpatient investigation. The scan demonstrated poor contrast opacification of the left innominate vein with prominent mediastinal and chest wall collateral vessels, suggestive of central venous thrombosis involving the left brachiocephalic (innominate) vein and raising the possibility of a chronic in situ process rather than acute embolic disease. No suspicious masses, lymphadenopathy, or radiological evidence of malignancy were identified. Following this incidental finding, the patient was referred to the hospital medical team for further evaluation and management.

**Video 2:** Repeat CTCAP performed one year later for evaluation of unexplained weight loss showed poor contrast distension of the left innominate vein, raising suspicion of venous thrombosis. Contrast-enhanced CTCAP performed one year later demonstrating poor contrast distension of the left innominate vein with prominent collateral circulation, suggestive of venous thrombosis. CTCAP: computed tomography scan of the chest, abdomen, and pelvis

The patient had a background history of atrial fibrillation treated with apixaban 5 mg twice daily, with adherence confirmed during medication review and no dose-reduction criteria present. He had previously undergone dual-chamber permanent pacemaker implantation (DDD mode) on March 14, 2025, via a left subclavian venous approach for symptomatic bradyarrhythmia, approximately 10 months before the diagnosis of venous thrombosis. Device interrogation demonstrated stable pacing parameters with appropriate sensing and pacing thresholds.

On hospital assessment, the patient was hemodynamically stable and afebrile. There were no clinical signs of upper extremity venous obstruction, including arm swelling, erythema, tenderness, dilated superficial veins, facial plethora, or features suggestive of superior vena cava syndrome. Cardiovascular examination demonstrated an irregular rhythm consistent with atrial fibrillation. There were no peripheral stigmata of infective endocarditis, and no lymphadenopathy or organomegaly was detected. Laboratory investigations performed during hospital evaluation were largely unremarkable apart from mild hyponatremia (132 mmol/L). Hemoglobin was 135 g/L, C-reactive protein 2 mg/L, estimated glomerular filtration rate 81 mL/min/1.73 m², and creatinine 75 µmol/L. Liver function tests, serum protein electrophoresis, and antineutrophil cytoplasmic antibodies were within normal limits (Table 1). D-dimer was mildly elevated (0.86 µg/mL), which is a non-specific finding in an elderly patient, but was considered consistent with the presence of venous thrombosis. Serum IgM was mildly reduced; however, isolated low IgM without other immunological abnormalities was considered clinically insignificant.

**Table 1:** Routine blood investigations on admission. WBC: white blood cell count; eGFR: estimated glomerular filtration rate; ALT: alanine aminotransferase; ALP: alkaline phosphatase; CRP: C-reactive protein; ESR: erythrocyte sedimentation rate; IgG: immunoglobulin G; IgA: immunoglobulin A; IgM: immunoglobulin M; TSH: thyroid-stimulating hormone; free T4: free thyroxine; ACE: angiotensin-converting enzyme

Parameter	Value	Units	Reference range
Creatinine	75	µmol/L	64-104 µmol/L
eGFR	81	mL/min/1.73 m²	>60
Bilirubin	13	µmol/L	0-29 µmol/L
ALT	41	U/L	0-70 U/L
ALP	66	U/L	30-130 U/L
Albumin	40	g/L	35-50 g/L
CRP	2	mg/L	0-10 mg/L
D-dimer	0.86	µg/mL	0.05-0.50 µg/mL
Phosphate	0.93	mmol/L	0.8-1.5 mmol/L
Serum ACE	39	U/L	20-70 U/L
Amylase	51	U/L	0-125 U/L
Sodium	132	mmol/L	133-146 mmol/L
Potassium	5.1	mmol/L	3.5-5.3 mmol/L
Hemoglobin	135	g/L	130-180 g/L
WBC	6.9	×10⁹/L	4.0-11.0 × 10⁹/L
ESR	2	mm/h	1-30 mm/h
IgG	8.3	g/L	6-16 g/L
IgA	3.3	g/L	0.8-4 g/L
IgM	0.42	g/L	0.5-2 g/L
TSH	1.5	mIU/L	0.4-5 mIU/L
Free T4	14	pmol/L	9-19 pmol/L
Vitamin B12	400	ng/L	189-883 ng/L
Folate	8.4	µg/L	3.1-20 µg/L

To further characterize the venous abnormality identified on CT imaging, targeted ultrasonography (Video 3) of the left subclavian vein at the pacemaker lead insertion site was performed. Ultrasound demonstrated a short-segment thrombus adjacent to the pacing leads, causing partial luminal occlusion with associated non-compressibility and absent Doppler flow extending approximately 3 cm proximally, findings consistent with lead-associated subclavian vein thrombosis.

**Video 3:** Targeted ultrasonography of the left subclavian vein at the pacemaker lead insertion site demonstrated a short-segment thrombus partially occluding the lumen, with associated non-compressibility and absent Doppler flow extending approximately 3 cm proximally, consistent with lead-associated subclavian vein thrombosis. Ultrasound of the left subclavian vein at the pacemaker lead entry site showing a short-segment thrombus abutting the pacing leads, partial luminal occlusion, non-compressibility, patchy hyperechogenicity, and absent Doppler flow over approximately 3 cm, consistent with subclavian vein thrombosis.

Despite confirmed therapeutic anticoagulation, no alternative provoking factors were identified. There was no clinical, biochemical, or radiological evidence of malignancy or infective endocarditis, and the patient had no prior history of venous thromboembolism or known inherited thrombophilia. Formal thrombophilia testing was not pursued during the initial evaluation because the presence of transvenous pacing leads represented a clear local provoking factor, and the event occurred in the setting of therapeutic anticoagulation. Similarly, tumor marker testing was not performed because cross-sectional imaging did not demonstrate suspicious lesions, and tumor markers are not recommended as screening tests for malignancy in the absence of clinical or radiological suspicion.

Following discussion with the cardiology team, anticoagulation was transitioned from apixaban to warfarin with temporary low-molecular-weight heparin bridging, allowing INR-guided monitoring. This approach reflected clinician preference in the context of thrombosis occurring during therapeutic anticoagulation rather than evidence of superiority of vitamin K antagonists over DOACs for device-associated thrombosis. Device extraction was not considered, as there was no evidence of device infection, malfunction, or clinically significant venous obstruction.

The patient was referred to the anticoagulation clinic for ongoing monitoring. As part of the continued evaluation for weight loss, a fecal immunochemical test (FIT) was arranged through primary care, and an outpatient gastroenterology review with planned endoscopic evaluation was organized. Follow-up venous ultrasonography at three months was scheduled to assess thrombus progression or resolution.

## Discussion

Pacemaker lead-associated venous thrombosis is a recognized but frequently underdiagnosed complication of transvenous CIEDs. Imaging studies have demonstrated that venous abnormalities following device implantation are relatively common. Venographic studies report venous stenosis or occlusion in approximately 23%-64% of patients; however, the majority of these findings remain clinically silent. In contrast, symptomatic UEDVT occurs in only around 1%-5% of patients with transvenous leads. This distinction between imaging-detected venous abnormalities and clinically significant thrombosis is important when interpreting prevalence data and assessing clinical relevance [10-12].

In the present case, multimodality imaging supported an in situ thrombotic process rather than embolic disease. CT demonstrated poor contrast opacification of the left innominate vein with prominent mediastinal and chest wall collateral vessels, suggesting a chronic venous obstruction. Targeted duplex ultrasonography subsequently confirmed thrombus adjacent to the pacemaker leads with partial luminal occlusion of the proximal subclavian vein. The incidental nature of this finding reflects a scenario increasingly encountered in contemporary clinical practice, where cross-sectional imaging performed for unrelated clinical indications leads to the detection of asymptomatic central venous thrombosis [13].

The pathophysiology of device-associated thrombosis is multifactorial and is predominantly driven by local mechanical and endothelial factors related to the presence of transvenous leads. Endothelial injury may occur during lead implantation and can persist due to chronic mechanical irritation of the venous wall by the intravascular device. In addition, the pacing lead acts as a foreign body within the venous circulation, altering laminar blood flow and creating areas of turbulence and relative venous stasis. These processes collectively contribute to a prothrombotic environment consistent with Virchow’s triad, including endothelial injury, abnormal blood flow, and activation of the coagulation cascade. Over time, this local prothrombotic milieu may promote thrombus formation adjacent to pacing leads and may result in partial venous obstruction or the development of collateral venous circulation.

Systemic anticoagulants such as vitamin K antagonists and DOACs primarily act by inhibiting components of the coagulation cascade and thereby reducing thrombin generation. However, these agents do not directly modify the underlying mechanical factors associated with indwelling intravascular leads. Consequently, thrombus formation can occur despite therapeutic anticoagulation in some patients with transvenous devices. Importantly, this observation should not be interpreted as evidence of anticoagulant treatment failure but rather reflects the complex interaction between systemic coagulation pathways and local device-related factors. At present, there are no randomized studies comparing anticoagulant strategies specifically for pacemaker lead-associated thrombosis [14-16].

Management strategies are therefore largely extrapolated from the broader venous thromboembolism literature. Current guidance for catheter- or device-related upper extremity venous thrombosis generally recommends anticoagulation for at least three to six months, with treatment duration tailored according to thrombus burden, symptom severity, and the presence of ongoing risk factors such as indwelling leads. Both vitamin K antagonists and DOACs have been used successfully in this context, and the choice of anticoagulant is typically guided by patient-specific clinical factors, bleeding risk, comorbidities, and clinician preference [17,18].

In the present case, anticoagulation was transitioned from apixaban to warfarin following specialist consultation. This decision reflected clinical judgment in the context of thrombosis detected despite therapeutic anticoagulation rather than evidence demonstrating the superiority of one anticoagulant strategy over another for device-related thrombosis. In clinical practice, vitamin K antagonists may occasionally be selected when closer monitoring of anticoagulation intensity is desired through measurement of the INR, allowing clinicians to assess therapeutic anticoagulation more directly. Follow-up imaging is frequently performed in such cases to assess thrombus stability or resolution and to guide the duration of therapy.

This case illustrates the diagnostic and therapeutic challenges associated with incidentally detected pacemaker lead-associated venous thrombosis and highlights the value of multimodality imaging in establishing the diagnosis. It also underscores the importance of careful clinical assessment when thrombosis occurs in patients already receiving therapeutic anticoagulation. In the absence of randomized data guiding anticoagulant selection in this setting, management decisions remain individualized and should involve multidisciplinary discussion with cardiology and thrombosis specialists.

Overall, this case highlights a common clinical dilemma encountered in acute and general medical practice rather than a rare phenomenon. Awareness of pacemaker lead-associated venous thrombosis and a structured, mechanism-based approach to anticoagulation decisions are essential, particularly for junior clinicians managing such findings during on-call settings (Interactive Model 1).

**Video 1:** 3D model: pacemaker lead venous thrombosis. This 3D model illustrates the anatomical relation between the transvenous pacemaker lead and the adjacent venous structures. This model was created by the authors.

## Conclusions

Pacemaker lead-associated central venous thrombosis is an under-recognized complication of transvenous cardiac device implantation and may be detected incidentally on cross-sectional imaging. Such thrombosis can occur in the absence of malignancy, infection, or systemic prothrombotic conditions, suggesting an important contribution from local device-related factors. Currently, there are no dedicated guideline recommendations specifically addressing anticoagulation strategies for pacemaker lead-associated thrombosis, and management is generally extrapolated from data on catheter-related or upper extremity venous thromboembolism. Consequently, treatment decisions remain individualized, taking into account thrombus extent, symptom burden, bleeding risk, and the underlying indication for anticoagulation.

Robust comparative evidence guiding the choice between vitamin K antagonists and DOACs in device-related thrombosis remains limited. Follow-up imaging at three to six months may be considered to assess thrombus stability or resolution and inform ongoing anticoagulation decisions, although this practice is largely based on expert opinion rather than definitive guideline recommendations. As a single case observation, this report cannot establish causality or superiority of any particular management strategy. Nevertheless, greater awareness of pacemaker lead-associated venous thrombosis and careful individualized follow-up may help clinicians recognize and manage this entity in patients with CIEDs.
